# Fungal Communities in Various Environments

**DOI:** 10.3390/jof11080560

**Published:** 2025-07-29

**Authors:** Nan Li, Ke Dong

**Affiliations:** 1 Key Laboratory of Climate, Resources and Environment in Continental Shelf Sea and Deep Sea of Department of Education of Guangdong Province, Department of Oceanography, Key Laboratory for Coastal Ocean Variation and Disaster Prediction, College of Ocean and Meteorology, Guangdong Ocean University, Zhanjiang 524088, China; 2 Major in Life Science, College of Convergence Science, Kyonggi University, 154-42, Gwanggyosan-ro, Yeongtong-gu, Suwon-si 16227, Gyeonggi-do, Republic of Korea

## 1. Introduction

Fungi are fundamental components of ecosystems that play indispensable roles in biomass decomposition and nutrient cycling. Through complex symbiotic and antagonistic interactions with flora, fauna, and diverse microbial communities, fungi contribute to the regulation of ecosystem balance and the maintenance of biodiversity [1,2,3]. Fungal communities display significant variability in both their taxonomic composition and functional potential across diverse environmental contexts and spatiotemporal scales. These variations underscore the adaptive strategies employed by fungi in response to specific environmental perturbations and offer valuable insights into how environmental changes affect ecosystem functioning, resilience, and stability [4,5,6,7].

This Special Issue comprises 18 original research articles that collectively advance our understanding of the fungal diversity, ecological functions, and mechanisms governing community assembly across a range of ecosystems. The contributions specifically examine fungal community responses to environmental gradients, including variations in soil type, nutrient availability, elevation, and vegetation composition. Furthermore, the studies elucidate the complex interactions between the biotic and abiotic factors that govern fungal diversity, co-occurrence networks, and functional traits. Spanning ecosystems from mangrove forests to temperate woodlands, and from rhizosphere microbiomes to high-elevation sediments, the studies encompass molecular to ecosystem-level analyses, providing a comprehensive exploration of fungal community dynamics. In addition to synthesizing the core findings of these contributions, this editorial aims to contextualize them within a broader ecological framework, thereby bridging established knowledge with emerging research. Notable examples include the application of long-read sequencing technologies and the investigation of the ecological significance of particular fungal lineages in ecosystem processes. Moving beyond a mere summary, this editorial seeks to provide an integrative perspective that is firmly grounded in prior research while highlighting future directions for advancing fungal ecological studies.

## 2. Overview of Published Articles

A group of studies in this Special Issue illuminated the responses of fungal communities to environmental variability across diverse ecosystems, offering insights from multiple ecological perspectives. For instance, Contribution 1 investigated fungal co-occurrence networks in clay versus sandy soils and found that networks in clayey soils exhibited significantly greater complexity and stability in mangrove ecosystems. Ammonium nitrogen (NH_4_^+^-N) and total nitrogen were identified as key drivers of the network structure. In clay soils, both fungal α-diversity and soil physicochemical properties jointly influenced the complexity and stability of communities, whereas in sandy soils, β-diversity played a more dominant role. Similarly, Contribution 2 examined the fungal community shifts during mangrove restoration and found that the dominant taxa and trophic modes differed significantly across successional stages. Although α-diversity remained relatively stable, β-diversity varied markedly. Total nitrogen and inorganic phosphorus strongly influenced α-diversity, while temperature and pH were key determinants of β-diversity. Trophic mode diversity was further driven by the total carbon, total nitrogen, and phosphorus fractions. Collectively, these findings highlight the central role of nutrient availability in shaping the structure and function of the fungal community. In forest ecosystems, Contribution 3 explored fungal functional dynamics during litter decomposition in a mixed plantation of Liquidambar formosana and Pinus thunbergii. The study reported that fungal functional guilds in mixed forests differ significantly from those in monoculture systems. The decomposition stage and litter type (leaf vs. twig) influenced enzymatic activity and nutrient-cycling gene abundance, with ectomycorrhizal fungi being more abundant in the mixed stands. In agricultural soils, Contribution 4 revealed that different *Morchella* species can distinctly restructure soil fungal communities and mineral nutrient availability through species-specific microbial and nutrient interactions. Functional shifts between the early and late stages of decomposition underscored the integrated effects of dominant tree species, litter quality, and decomposition time on fungal community composition and associated processes. Furthermore, Contribution 5 documented the contrasting responses of foliar endophytic fungi and bacteria to thinning intensity within temperate forest phyllosphere ecosystems. Fungal diversity and the complexity of co-occurrence networks increased in response to thinning; however, shifts in microbial communities were predominantly driven by indirect effects. Specifically, thinning altered the diversity of neighboring trees, which subsequently modified leaf traits, such as the specific leaf area and dry matter content, ultimately reshaping endophyte fungal assemblages.

Elevation gradients have emerged as consistent determinants of fungal community dynamics. For example, Contribution 6 reported divergent elevational trends in fungal diversity across three mountain ranges on the Korean Peninsula, each situated within a different climatic zone, underscoring the pronounced sensitivity of fungal communities to environmental gradients. Contribution 7 showed that in both the rhizosphere and root endosphere of the endangered Heptacodium miconioides, arbuscular mycorrhizal fungal (AMF) colonization rates and spore densities increased with elevation, whereas internal AMF diversity concomitantly declined. Contribution 8 further demonstrated that elevation-induced changes in the fungal community structure affected microbial residual carbon and soil organic carbon (SOC) stabilization, suggesting that the SOC retention capacity may decline in high-elevation environments due to shifts in fungal assemblages. Contribution 9 examined the elevational responses of fungal communities along the Yellow River in sedimentary ecosystems. They observed a decline with regard to the diversity with increasing altitude, whereas the co-occurrence networks became more complex and positively interactive at higher elevations. Community assembly processes are dominated by deterministic forces, particularly homogeneous selection, at higher elevations.

Rhizosphere microbiomes, as critical mediators of plant–soil interactions, are shaped by plant identity, developmental stage, and edaphic variables. Contribution 7 identified soil pH, available phosphorus, and total nitrogen as the main drivers of rhizosphere AMF composition, whereas internal AMF communities were more responsive to nitrate, available potassium, and acid phosphatase activity. Contribution 10 compared the rhizosphere microbiota of cultivated wheat and its wild progenitor (Triticum dicoccoides) in agricultural systems and revealed largely conserved community structures, although cultivated wheat harbored more fungal pathogens, greater functional redundancy, and the enhanced enrichment of carbon and nitrogen cycling genes. In an urban context, Contribution 11 investigated ancient versus mature Ginkgo biloba trees in Shanghai and showed that ancient trees supported richer rhizosphere AMF communities dominated by Paraglomus and Glomus, with a higher abundance of viable AMF propagules. These findings emphasize the influence of pH and phosphorus on community assembly. Similarly, Contribution 12 studied eucalyptus plantations of varying stand ages in southern Jiangxi, noting shifts in AMF community composition across successional stages, driven by phosphorus, nitrogen, and soil bulk density.

Several studies have investigated the diversity and the ecological roles of specific fungal taxa in distinct ecosystems. For instance, Contribution 13 documented 88 species of Agaricomycetes wood-decaying fungi across the Amazonian floodplain forest islands, revealing pronounced community differentiation among the islands and underscoring the region’s exceptional fungal diversity and conservation significance.

Moreover, Contribution 14 identified multiple previously unrecorded Myxomycetes species in Jilin Province, underscoring their importance in regional biodiversity surveys, while the pathogenic fungi associated with plants and arthropods also represented a significant area of focus. Contribution 15 identified Neofusicoccum parvum and Diaporthe spp. as key agents in complex pomegranate wood canker diseases, indicating multifactorial interactions between fungi and abiotic stressors. In the Angeles National Forest, Contribution 16 recorded 14 Phytophthora species, including the first report of P. taxon oakpath (subclade 8e), in both symptomatic and asymptomatic plants, especially near water sources—raising concerns about cryptic Phytophthora diversity and the risk of disease in drought-stressed Californian ecosystems. Contribution 17 describes *Gibellula floridensis*, a novel spider-pathogenic species identified in the subtropical habitats of north–central Florida. Their findings revealed broad yet habitat-dependent distributions of entomopathogenic fungi, prompting further inquiries into their occurrence, prevalence, and potential ecological functions beyond their interactions with arthropod hosts.

Finally, the rapid advancement of high-throughput sequencing technologies has substantially improved the resolution and scalability of fungal community analysis. Contribution 18 assessed the applicability of nanopore sequencing to fungal metabarcoding using ectomycorrhizal fungi in decayed *Fagus sylvatica* bark. The study proposed an “eNano” workflow and compared two taxonomic assignment strategies: reference matching based on the UNITE Species Hypotheses (SH) framework and de novo OTU clustering at 98% similarity. Both approaches effectively processed Nanopore data, with SH offering higher cross-study comparability, and OTU-based clustering performing better under Q ≥ 25 thresholds. Ecological analysis revealed that the degree of wood decay significantly shaped the ectomycorrhizal community structure, with Laccaria amethystina and Tomentella sublilacina dominating the root tips of decayed logs. This study not only confirmed the feasibility of nanopore sequencing in fungal ecology but also demonstrated the role of decay-driven niche partitioning in forest ecosystems.

## 3. Outlook and Prospects

As the research on fungal communities across diverse ecosystems continues to advance, our understanding of their diversity patterns, environmental response mechanisms, and ecological functions has grown substantially. However, several conceptual and methodological bottlenecks remain and new frontiers are rapidly emerging. First, both spatial and temporal scales of fungal ecological research require substantial expansion. Most current studies offer only a narrow view that constrains our understanding of broad-scale biogeographic patterns and long-term temporal dynamics [8]. Future research should prioritize long-term ecological monitoring and large-scale multisite comparisons to integrate macroecological processes with microbial-scale responses, thereby uncovering the spatiotemporal dynamics of fungal community assemblies and their contributions to ecosystem functionality. Second, greater emphasis must be placed on community-level functional attributes, because the transition from taxonomic diversity to functional trait-based approaches has emerged as a central theme in modern ecology. However, the fundamental aspects of fungal community function, such as functional redundancy, niche partitioning, and mechanistic coupling with carbon, nitrogen, and phosphorus cycling, remain insufficiently characterized [9,10,11,12]. Systematic experimental validation and theoretical modeling are essential for developing a coherent framework that links fungal community traits to biogeochemical processes [13,14]. Parallel efforts must focus on improving the annotation and empirical verification of putative functional genes in order to reduce dependence on computational predictions lacking experimental substantiation. Third, technological innovations continue to open new avenues for fungal ecological studies. Long-read sequencing platforms (e.g., Nanopore and PacBio) and single-cell omics provide enhanced taxonomic resolution and enable the detection of rare or cryptic taxa in complex environments [15,16]. Coupled with metabolomics, stable isotope tracing, and ecological stoichiometry, these approaches support a critical transition from merely identifying community composition (“who is there”) to elucidating functional roles and activities (“what they are doing”). Such integration approaches are essential to develop a mechanistic understanding of the roles of fungi in ecosystems. Moreover, fungal ecology must be situated within a broader interdisciplinary framework because interactions between fungi and other organisms, including plants, animals, bacteria, and viruses, are fundamental for maintaining ecosystem resilience and network stability. Incorporating fungi into “multihabitat, multikingdom” interaction networks is crucial for achieving a systems-level perspective on ecological equilibrium. This integration is particularly urgent in the context of global environmental change because extreme climatic events, land-use transformations, and biological invasions may drive nonlinear or threshold responses in fungal communities, with cascading effects on ecosystem functions [17,18,19,20,21]. Balancing fungal bioprospecting with ecological conservation warrants further investigation. Numerous studies have highlighted the rich and often unique fungal diversity in sensitive environments such as tropical rainforests, mangroves, and sedimentary habitats. These taxa have untapped potential for applications in medicine, agriculture, and environmental remediation [22,23,24,25]. However, any exploitation of these resources must be preceded by rigorous assessments of their ecological vulnerability and sustainability, ideally embedded within risk-controlled conservation frameworks.

We anticipate that future fungal ecology will be increasingly defined through cross-scale, cross-system, and cross-disciplinary integration. Such approaches are essential for transitioning the field from foundational biodiversity assessments to deeper mechanistic explanations of ecosystem processes, ultimately supporting sustainable management and policy development in a changing world.
